# Characterization of AcrD, a Resistance-Nodulation-Cell Division-type multidrug efflux pump from the fire blight pathogen *Erwinia amylovora*

**DOI:** 10.1186/1471-2180-14-13

**Published:** 2014-01-21

**Authors:** Daniel Pletzer, Helge Weingart

**Affiliations:** 1 School of Engineering and Science, Jacobs University Bremen, Campus Ring 1, 28759 Bremen, Germany

**Keywords:** Plant pathogen, Fire blight, *Erwinia amylovora*, Multidrug efflux, RND transporter, AcrD

## Abstract

**Background:**

Multidrug efflux pumps are membrane translocases that have the ability to extrude a variety of structurally unrelated compounds from the cell. AcrD, a resistance-nodulation-cell division (RND) transporter, was shown to be involved in efflux of highly hydrophilic aminoglycosides and a limited number of amphiphilic compounds in *E. coli*. Here, a homologue of AcrD in the plant pathogen and causal agent of fire blight disease *Erwinia amylovora* was identified.

**Results:**

The substrate specificity of AcrD was studied by overexpression of the corresponding gene from a high-copy plasmid in *E. amylovora* Ea1189-3, which is hypersensitive to many drugs due to a deficiency of the major multidrug pump AcrB. AcrD mediated resistance to several amphiphilic compounds including clotrimazole and luteolin, two compounds hitherto not described as substrates of AcrD in enterobacteria. However, AcrD was not able to expel aminoglycosides. An *acrD* mutant exhibited full virulence on apple rootstock and immature pear fruits. RT-PCR analysis revealed an induction of *acrD* expression in infected apple tissue but not on pear fruits. Moreover, a direct binding of BaeR, the response regulator of the two-component regulatory system BaeSR, to the *acrD* promoter was observed as has already been shown in other enterobacteria.

**Conclusions:**

AcrD from *E. amylovora* is involved in resistance to a limited number of amphiphilic compounds, but in contrast to AcrD of *E. coli*, it is not involved in resistance to aminoglycosides. The expression of *acrD* was up-regulated by addition of the substrates deoxycholate, naringenin, tetracycline and zinc. AcrD appears to be regulated by the BaeSR two-component system, an envelope stress signal transduction pathway.

## Background

*Erwinia amylovora* is the causative agent of fire blight, a destructive, contagious disease of apple, pear, and other rosaceous plants [1]. All aerial parts of the hosts can be infected by the pathogen. *E. amylovora* enters its host plants through natural openings (e.g., flower nectaries or leaf stomata) and wounds [2]. Upon entry, the fire blight pathogen moves through intercellular spaces towards the xylem [3]. Typical symptoms include flower necrosis, immature fruit rot, shoot curvature (shepherd’s crook), bacterial ooze secretion, and cankers on woody tissues [1]. The most effective method to treat infected plants is pruning to remove all infected tissue.

However, fire blight can infect entire orchards within a single growing season leading to devastating economic losses [4]. Presently, there are no effective therapeutics available to cure fire blight and therefore prevention is considered the best solution to manage this plant disease. Current control efforts are rather rare and rely primarily on antibiotic applications (e.g., streptomycin or oxytetracycline) to protect flowers. However, the use of antibiotics for the management of fire blight is highly controversial due to the potential risk of promoting the emergence and spread of antibiotic resistance [5].

Gram-negative bacteria often possess multidrug efflux transporters within the cytoplasmic membrane, which have been found to recognize and expel a broad range of structurally unrelated compounds from the cell [6,7]. Among the multidrug efflux pumps, members of the resistance-nodulation-cell division (RND) family appear to be the most effective efflux systems in Gram-negative bacteria. RND transporters form a tripartite complex, consisting of an inner membrane pump that recognizes and captures the substrates, a periplasmic membrane fusion protein (MFP) and an outer membrane channel [8,9].

AcrAB is the major multidrug efflux pump in *E. coli* and shows high conservation among Gram-negative bacteria [8,10-12]. AcrD, a close homolog of AcrB, is an RND-type efflux pump characterized as a transporter of aminoglycosides, a highly hydrophilic class of molecules, and as a transporter of several amphiphilic compounds [13,14]. Typically, the inner membrane pump and the periplasmic MFP are co-transcribed in tandem on polycistronic mRNA molecules [15]. Interestingly, this is not the case for *acrD*, which appears as a single gene and seemingly functions in concert with AcrA, a MFP co-transcribed with AcrB [14].

Both AcrAB and AcrD efflux systems are also present in the plant pathogen *E. amylovora*. AcrAB has already been characterized as an efflux system required for virulence of *E. amylovora* in resistance towards apple phytoalexins and for successful colonization of the host plant [16]. AcrAB of *E. amylovora* showed a similar substrate spectrum as AcrAB of *E. coli*[17]. In this study, the substrate specificity of AcrD from *E. amylovora* was characterized and its contribution to virulence in apple and pear analyzed. As it was found that *acrD* is expressed only at low levels under *in vitro* conditions, we were interested in investigating whether the expression of the AcrD transporter in *E. amylovora* is induced *in planta*.

Multidrug transporters are often expressed under control of local, as well as, global transcriptional regulators [18]. Current data show that expression of *acrD* in *E. coli* can be induced by the two-component regulatory system BaeSR [19]. Two-component systems (TCS) play an important role in the regulation of physiological processes in response to environmental or cellular parameters and enable bacterial cells to adapt to changing environmental conditions. TCSs typically consist of a membrane-bound histidine protein kinase (HPK) whose autokinase activity is dependent upon sensing a specific environmental stimulus (e.g., nutrients, temperature, pH, toxins or oxidative stress) [20]. The second protein of a TCS is a response regulator, onto which a phosphoryl group is transferred from the phosphorylated HPK, and which functions as a phosphorylation-activated switch that regulates output responses within the cell causing changes in the expression of target genes [21,22].

BaeSR is a TCS that responds cell envelope damages in *E. coli*[23]. The small core regulon of BaeSR includes the RND-type transporters AcrD and MdtABC and the periplasmic chaperone Spy [24]. The presence of a homologous BaeSR system in *E. amylovora*, prompted us to analyze the impact of the response regulator BaeR on the expression levels of *acrD*.

Herein, we report that overexpression of the RND pump AcrD in an *acrB*-deficient mutant leads to increased resistance to two substrates, clotrimazole and luteolin, previously not described as substrates of AcrD in other enterobacteria. In order to determine the promoter activity *in vitro*, we utilized a transcriptional fusion of the promoter regions of *acrAB* and *acrD*, respectively, with the reporter gene *egfp*. We demonstrate that the response regulator BaeR is able to bind to the upstream region of *acrD* in *E. amylovora* Ea1189 and to induce *acrD* expression. Furthermore, we show that the inactivation of the RND pump AcrD did not result in reduction of virulence of *E. amylovora* on host plants.

## Results

### Identification of an *acrD* homologue in *E. amylovora* Ea1189

A search with the BLASTP program (NCBI) using the amino acid sequence of AcrD from *E. coli* K-12 as the query (accession number P24177) identified a homologous sequence in the genome of *E. amylovora* CFBP1430 (GenBank:EAMY_2508, annotated as *cmeB*). The annotated protein EAMY_2508 is 18 amino acids shorter at the N-terminus than the AcrD protein of *E. coli*. Comparison of the *acrD* gene sequences from *E. coli* and *E. amylovora* suggests that the EAMY_2508 gene of *E. amylovora* CFBP1430 has been annotated with a wrong start codon. Sequence analysis revealed an alternative ATG start codon 54 bp upstream of the annotated EAMY_2508 gene. The 18 amino acids, encoded by the 54 additional nucleotides, are 100% identical to the N-terminal region of AcrD from *E. coli*.

We used the genome sequence of *E. amylovora* CFBP1430 to design primers to PCR amplify the respective region from the genomic DNA of our model strain *E. amylovora* Ea1189 whose genome sequence is not yet available. The nucleotide sequence of *acrD* and its upstream region from *E. amylovora* Ea1189 show 100% identity to EAMY_2508 and its upstream region from *E. amylovora* CFBP1430 based on our sequencing results. AcrD is a member of the RND superfamily of transporters belonging to the Hydrophobe/Amphiphile Efflux-1 (HAE1) family (Transporter Classification Database TC #2.A.6.2.7).

A BLASTP search (NCBI) of the deduced AcrD sequence from *E. amylovora* Ea1189 as query identified homologous multidrug efflux transporters with high degree of sequence identity (78 - 95%) in several members of the *Enterobacteriaceae* family (see Additional file 1 for a phylogenetic tree).

A sequence alignment between AcrD from *E. amylovora* Ea1189 and AcrD from *E. coli* K-12 showed that the proteins share 79% identity and 89% similarity with each other (see Additional file 2). Substituted amino acids were distributed throughout the sequence, but they were at least 40% conserved (all substitutions show a physico-chemical score of minimum 4) [25-28] and no insertion or deletion was observed.

Analysis of the up- and downstream regions flanking the *acrD* homologues from *E. amylovora*, *E. coli* and *S. enterica* revealed several differences (see Additional file 3) including the two-component system NarQP located upstream of *acrD* in *E. amylovora*. This system is involved in the regulation of anaerobic nitrate/nitrite respiration, and consists of the sensor kinase NarQ and the response regulator NarP. In *E. coli* and *S. enterica*, only the sensor kinase NarQ is present upstream of *acrD*. The response regulator NarP is situated at different positions in the genomes of *E. coli* and *S. enterica*. Moreover, the sizes of the NarQ homologues are also disparate. NarQ of *E. amylovora* Ea1189 is a protein consisting of 328 amino acids, whereas the NarQ homologues of *E. coli* and *S. enterica* consist of 566 amino acids.

The downstream region of *acrD* of *E. amylovora* Ea1189 contains an insertion of about 1.5 kb encoding several small hypothetical proteins.

### Transmembrane organization of AcrB and AcrD in *E. amylovora*

In a previous study, the transmembrane organization of AcrB and AcrD from *E. coli* was analyzed *in silico*, with 12 transmembrane-spanning domains (TMD) and 2 large periplasmic loops predicted in both proteins [14]. A similar approach was accomplished with AcrB and AcrD from *E. amylovora* Ea1189 using the online tool TOPCONS [29]. Topology analysis predicted the typical 12 TMDs and 2 periplasmic loops between TMD1 and 2 and TMD 7 and 8 for the RND-type efflux pumps AcrB and AcrD from *E. amylovora* Ea1189 (see Additional file 4).

### Phenotypic characterization of the *acrD* mutant

To evaluate the role of AcrD in antibiotic resistance and to identify substrates of this RND-type efflux pump, susceptibility tests of the wild type and the *acrD* mutant to a variety of antimicrobial agents were performed. Deletion of *acrD* resulted in no significant changes in sensitivity to tested aminoglycosides, dyes or detergents. However, the *acrD* mutant was 2-fold more sensitive to nitrofurantoin, erythromycin, silver nitrate and sodium tungstate in comparison to the wild type (Table 1). The differences in sensitivity were minor but reproducible. Complementation of the *acrD* mutant with plasmid pBlueKS.acrD, which carried the *acrD* gene of Ea1189 under control of the P_*lac*_, restored resistance to all tested antimicrobials (data not shown).

**Table 1:** **Antimicrobial susceptibility profiles from an *E. amylovora *** **wild-type strain, *acrD *** **mutant, *acrB *** **mutant complemented with AcrD-overexpression plasmids pBlueSK.acrD (*acrD *** **under control of P**_**lac**_**) and pBlueKS.acrD-ext (*acrD *** **under control of P**_**lac **_**and its native promoter P**_**acrD**_**) and *acrB *** **mutant complemented with control plasmid pBlueSK.acrD (*acrD *** **in opposite orientation to P**_**lac**_**)**

**Drug**	**MIC (μg/ml)**^***a***^				
	**Ea1189**	**Ea1189.acrD**	**Ea1189-3 (pBlueSK.acrD)**	**Ea1189-3 (pBlueKS.acrD)**	**Ea1189-3 (pBlueKS.acrD-ext)**
			**P**_**lac**_ **< < *acrD***	**P**_**lac **_**> >** ***acrD***	**P**_**lac**_**, P**_**acrD **_**> >** ***acrD***
**Antimicrobials**					
Benzalkonium chloride	12.5	12.5	1.2	1.2	ND
Chloramphenicol	3.1	ND	1.2	1.2	1.2
Clotrimazole	> 1000	> 1000	6.2	**12.5**	**25**
Fusaric acid	500	500	500	500	500
Fusidic acid	250	250	3.1	**6.2**	**25**
Genistein	> 5000	> 5000	62.5	62.5	62.5
Josamycin	125	125	3.1	3.1	3.1
Luteolin	> 5000	> 5000	15.63	15.6	**125**
Naladixic acid	2.5	2.5	1.2	1.2	1.2
Naringenin	5000	5000	312	312	312
Nitrofurantoin	25	**12.5**	12.5	12.5	12.5
Norfloxacin	0.63	0.63	0.03	0.03	0.03
Novobiocin	250	250	6.2	**25**	**100**
Phloretin	5000	5000	625	625	625
Rifampicin	12.5	12.5	12.5	12.5	12.5
Tetracycline	1.5	1.5	1.2	1.2	1.2
**Aminoglycosides**					
Amikacin	2.5	2.5	2.5	2.5	2.5
Gentamicin	2.5	2.5	2.5	2.5	2.5
Hygromycin B	100	100	62.5	**125**	**125**
Streptomycin	2.5	2.5	2.5	2.5	2.5
Tobramycin	2.5	2.5	2.5	2.5	2.5
**Macrolids**					
Azithromycin	0.31	0.31	0.63	0.63	0.63
Clarithromycin	0.31	0.31	0.31	0.31	0.31
Erythromycin	0.63	**0.31**	0.16	0.16	0.16
Roxithromycin	1.25	1.25	0.16	0.16	0.16
**Heavy metals**					
Cadmium acetate	12.5	12.5	25	**50**	**50**
Cobalt (II) chloride	625	625	1250	1250	1250
Copper (II) sulfate	1250	1250	1250	1250	1250
Nickel (II) chloride	1250	1250	2500	2500	2500
Silver nitrate	12.5	**6.2**	6.2	6.2	6.2
Sodium tungstate	125000	**62500**	125000	125000	125000
Zinc sulfate	156	156	156	**312**	**312**
					
**Dyes**					
Acriflavine	50	50	6.2	6.2	6.2
Crystal violet	3.1	3.1	2.5	2.5	2.5
Ethidium bromide	250	250	3.1	3.1	**6.2**
Rhodamine 6G	> 100	> 100	3.1	3.1	3.1
**Detergents**					
Bile salt	5000	5000	625	**1250**	**5000**
Deoxycholate	> 1000	> 1000	312	**1250**	**2500**
SDS	> 1000	> 1000	62.5	**125**	**125**

^*a*^MIC values were determined by the 2-fold dilution assay in three or more independent experiments with similar results. Boldface numbers indicate a higher or lower MIC. ND, not determined.

### Expression of *acrD* in an *acrB*-deficient mutant of *E. amylovora*

To investigate the substrate specificity of AcrD from Ea1189, overexpression of the corresponding gene from a high-copy plasmid was achieved in *E. amylovora* mutant Ea1189-3, which is hypersensitive to many drugs due to a deficiency of the major multidrug efflux pump AcrB [16]. Three overexpression plasmids were generated: pBlueKS.acrD, expressing *acrD* under control of the *lac* promoter (P_lac_), pBlueSK.acrD-ext, expressing *acrD* under control of its native promoter (P_acrD_) and pBlueKS.acrD-ext, expressing *acrD* under control of both promoters P_lac_ and P_acrD_. As a control, a promoterless *acrD* gene was cloned in the opposite direction of P_lac_. These plasmids were mobilized into the *acrB*-deficient mutant Ea1189-3 and the sensitivity of the transformants to various substrates were determined (Table 1). Ea1189-3(pBlueKS.acrD), expressing *acrD* under control of P_lac_, exhibited elevated resistance to clotrimazole (2-fold), fusidic acid (2-fold), novobiocin (4-fold), hygromycin B (2-fold), cadmium acetate (2-fold), zinc sulfate (2-fold), bile salt (2-fold), deoxycholate (4-fold), and SDS (2-fold). The expression of *acrD* under control of its native promoter in Ea1189-3 showed an increase in resistance similar to that of P_lac_-controlled *acrD* expression (data not shown). When *acrD* was under control of both promoters, P_lac_ and P_acrD,_ it conferred elevated resistance. Compared to the control, Ea1189-3(pBlueKS.acrD-ext) displayed increased resistance to clotrimazole (4-fold), fusidic acid (8-fold), novobiocin (16-fold), hygromycin B (2-fold), cadmium acetate (2-fold), zinc sulfate (2-fold), bile salt (8-fold), deoxycholate (8-fold), SDS (2-fold), luteolin (8-fold) and ethidium bromide (2-fold) (Table 1).

### RND-type efflux pump expression during cellular growth

**Figure 1:**
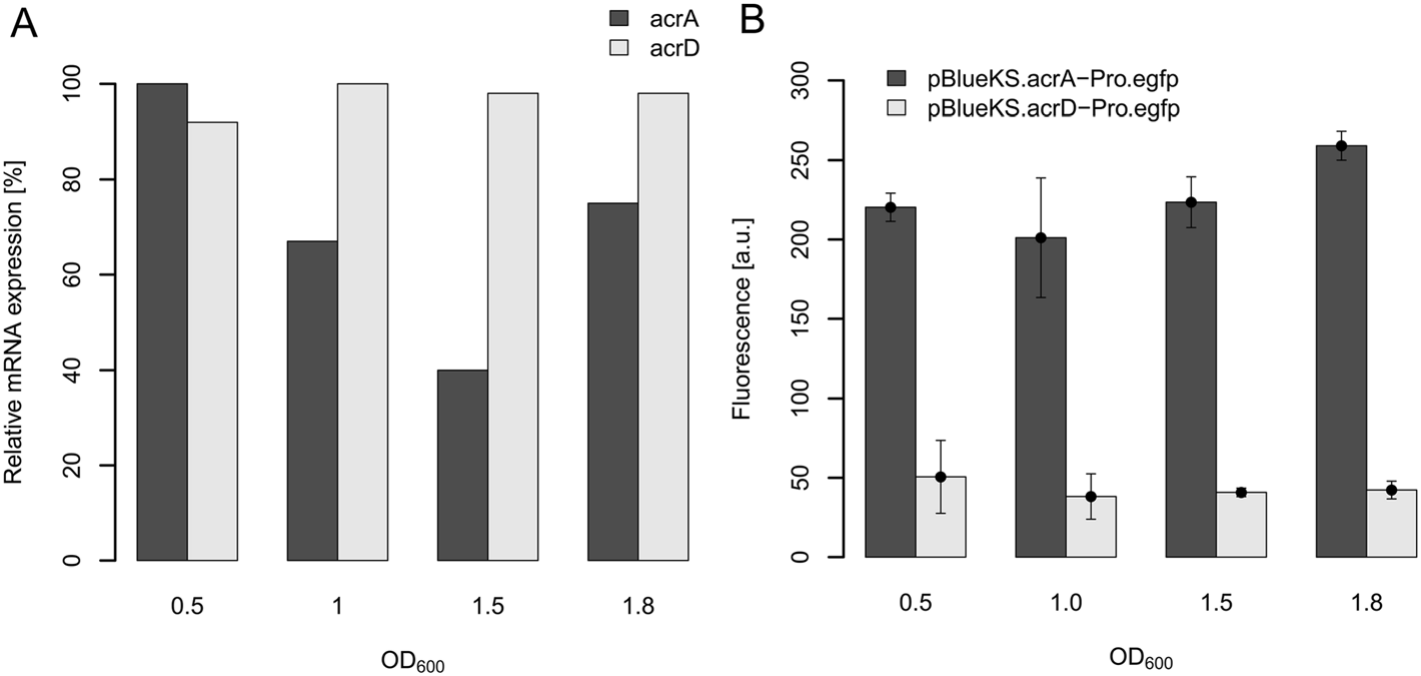
**Promoter activities of *acrA *and *acrD *from *Erwinia amylovora.*** The activity was determined in the course of growth in LB broth, OD_600_, optical density at 600 nm. **(A)** Relative mRNA transcript abundance of *acrA* and *acrD* during cellular growth of Ea1189 as determined by quantitative RT-PCR. The relative mRNA level was related to the highest average value determined for a gene, which was defined as 100%. **(B)** Expression of *acrA* and *acrD* as determined by transcriptional fusions with the reporter gene *egfp. E. amylovora* wild type was transformed with pBBR.acrA-Pro.egfp and pBBR.acrD-Pro.egfp, respectively. Experiments were performed in triplicates with similar results.

To monitor the expression levels of the RND-type efflux pumps AcrAB and AcrD at different growth states, total RNA was isolated at distinct optical densities and expression levels analyzed by quantitative RT-PCR. The expression values were normalized to the highest expression of the *acrA* and *acrD* transcript, respectively (Figure 1A). While the expression levels of *acrA* changed during the cell cycle, indicating a growth phase-dependent transcription with the highest expression in the early exponential phase, *acrD* showed constant expression during growth. Additionally, the constant expression of *acrD* was also connected to a low expression level as determined by C_t_ values (data not shown).

Furthermore, we studied the effect of temperature on activation of the RND-type efflux pump AcrD using qRT-PCR. Bacteria were cultured in LB broth at 18°C and 28°C, respectively, where 28°C represents the optimal growth temperature and 18°C represents the temperature at which several genes involved in pathogenicity showed induction in *E. amylovora*[30,31]*.* However, no temperature dependence of the *acrD* expression was observed *in vitro* (data not shown).

### Promoter activity of *acrAB* and *acrD in vitro*

In order to monitor promoter activities of the RND-type efflux pumps AcrAB and AcrD in *E. amylovora*, transcriptional fusions of the *acrA* upstream region (140 bp) and *acrD* upstream region (210 bp), respectively, to the enhanced green fluorescence protein-encoding gene (*egfp*) were constructed. To determine whether bacterial growth influenced the promoter activity, fluorescence measurements at several optical densities were performed (Figure 1B). Our data indicated that the promoter activities of both *acrAB* and *acrD* were constant throughout the growth phases in LB broth. Furthermore, the activity of the *acrD* promoter was 4 to 5-fold lower than the activity of the *acrAB* promoter throughout growth.

### Effect of substrate exposure on *acrD* expression

**Figure 2:**
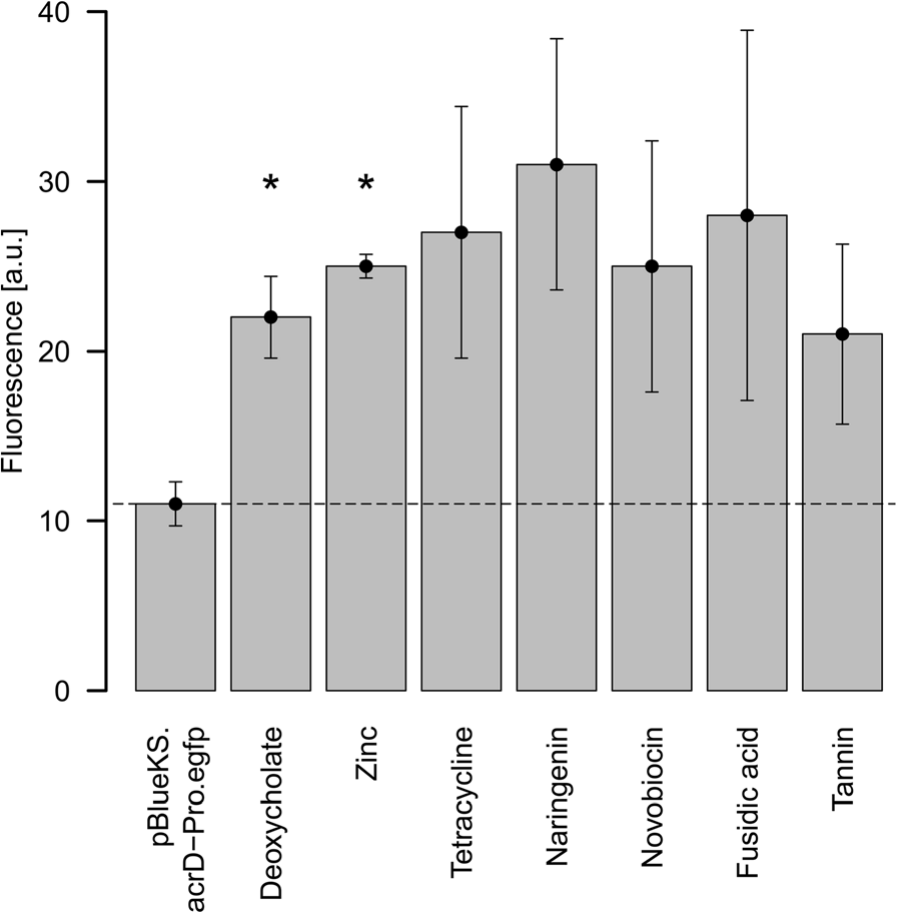
**Promoter activity of *acrD *from *Erwinia amylovora *determined by transcriptional fusions with the reporter *egfp*.** Fluorescence was determined 24 h after incubation of the bacteria with various transporter substrates. Substrates were added to a final concentration of 1:10 of the determined MIC values; deoxycholate (50 μg/ml), zinc sulfate (15.6 μg/ml), tetracycline (0.16 μg/ml), naringenin (31.2 μg/ml), novobiocin (1.2 μg/ml), fusidic acid (0.31 μg/ml) and tannin (500 μg/ml). The dotted line indicates the basal *acrD* promoter activity. Statistically significant differences (P < 0.05) are indicated by asterisks (*) and were determined by a two-sided *t*-test with equal variances. a.u., arbitrary units.

The expression of genes encoding multidrug efflux systems can be influenced by substrates, which interact with regulatory proteins and therefore increase gene transcription [32]. Above results prompted us to investigate whether antimicrobials affect the expression of the *acrD* gene in *E. amylovora*. Therefore, we utilized a transcriptional fusion between the promoter region of *acrD* and *egfp* (pBBR.acrD-Pro.egfp). In order to determine the promoter activity of *acrD*, we developed a screening assay in a 96-well-plate format. Antimicrobial compounds were added to the plasmid-harboring cells by the 2-fold dilution method and EGFP fluorescence was determined after 24 hours. Only fluorescence values from substrate concentrations that did not inhibit bacterial growth were plotted versus optical density on a scatter plot (see Additional file 5). Outliers, showing higher fluorescence than the remaining dataset, thus potential inducers of *acrD* expression, were identified as deoxycholate, naringenin, tetracycline and zinc sulfate. In the next step, the effect on the activity of the *acrD* promoter was evaluated in batch cultures. We included novobiocin and fusidic acid since they were identified as substrates of AcrD in *E. coli*[14,33]. Additionally, we tested tannin because it displayed a 2-fold induction of *acrD* in qRT-PCR analysis (data not shown). After 24 hours incubation, the fluorescence signal was measured and normalized to an OD_600_ of 0.1 (Figure 2). The tested substrates were able to induce the *acrD* promoter by approximately 2 to 3-fold. Among the tested substrates, deoxycholate and zinc, showed significant differences in comparison to the control (P < 0.05).

### Contribution of AcrD to virulence of *E. amylovora* on apple rootstocks

To study the impact of AcrD on virulence of *E. amylovora* Ea1189, apple rootstocks MM 106 were infected and the development of disease symptoms was monitored. After one week of incubation all infected shoots showed typical disease symptoms including the shepherd’s crook-like bending of the shoot tip, tissue necrosis and ooze formation surrounding the infection site. Furthermore, bacterial populations were counted 1 and 5 day(s) post inoculation, respectively. However, no significant differences between the populations of the wild type and the mutant were observed (Table 2).

**Table 2:** Virulence assay on apple rootstock MM 106

**Strain**	**Re-isolated bacterial cells**^***a***^	
	**1 dpi**	**5 dpi**
Ea1189	2.5 × 10^6^ ± 1.1 × 10^6^	4.7 × 10^8^ ± 1.1 × 10^8^
Ea1189.acrD	6.1 × 10^6^ ± 4.7 × 10^6^	3.5 × 10^8^ ± 1.1 × 10^8^

^*a*^Bacteria were inoculated by prick technique in the shoot tips with an inoculum of 5 × 10^6^ CFU/shoot. Establishment of a population of *Erwinia amylovora* Ea1189 and *acrD* mutant (CFU/shoot) was determined 1 and 5 days post inoculation (dpi), respectively.

**Figure 3:**
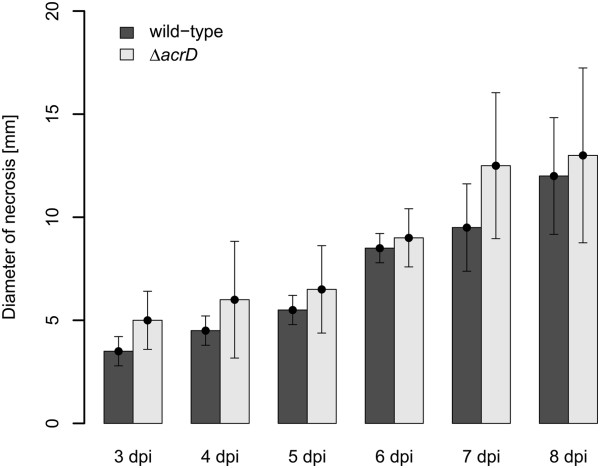
**Virulence of *Erwinia amylovora *Ea1189 wild type and the *acrD*-deficient mutant on immature pear fruits.** Symptoms were monitored starting from the 3^rd^ day post inoculation (dpi) until the fruits were completely necrotic (around 8 dpi). Data values represent the means of 6 replicates ± standard deviation.

Additionally, immature pear fruits were infected with the wild type and the *acrD*-deficient mutant and disease symptoms were monitored by means of the diameter of necrotic tissue surrounding the infection site (Figure 3). After 8 days of incubation, when the pear fruit was almost completely necrotic, no significant differences between the wild type and the mutant were observed.

### Transcriptional analysis of *acrA* and *acrD* of *E. amylovora in planta*

In order to analyze the *acrA* and *acrD* promoter activities *in planta*, Ea1189 was infected into shoot tips of apple rootstocks MM 106 as well as into immature pear fruits. Several hours (pears) and days (apple shoots), respectively, after inoculation bacteria were re-isolated by macerating infected plant areas. Total RNA was isolated from recovered cells and transcript abundances of *acrA* and *acrD* were determined by quantitative RT-PCR. RT-PCR signals of recovered bacteria were compared with RT-PCR signals of Ea1189 cells grown in LB broth to an OD_600_ of 0.5.

For immature pear infections, we first determined the expression of the sigma factor HrpL, which coordinates the transcription of genes of the hypersensitive response and pathogenicity (hrp) type III secretion system in *E. amylovora*, to identify the time of maximal expression of plant-inducible *hrp* genes. Compared to growth in LB broth, *hrpL* expression was 9-fold higher 6 h post inoculation into immature pears; after 12 h, expression was greatest (40-fold induction), and after 18 h, *hrpL* expression decreased again (10-fold induction). Hence, we investigated the expression of *acrA* and *acrD* genes with Ea1189 cells recovered from infected immature pear fruits 12 h after inoculation and compared them with cells grown in LB broth to an OD_600_ of 0.5 (Table 3). Our results indicated that neither *acrA* nor *acrD* are induced in the early infection phase of immature pear fruits.

**Table 3:** **Relative fold-changes in mRNA transcripts of *acrA *** **and *acrD *** **after inoculation of *Erwinia amylovora *** **Ea1189 on apple rootstocks MM106 and immature pear fruit slices, respectively**^*a*^

**Gene**	**Apple rootstock**			**Immature pear**
	**1 dpi**^***b***^	**4 dpi**	**7 dpi**	**12 hpi**^***c***^
*acrA*	-6.9	-5.8	-10.4	1.2
*acrD*	3.9	3.5	3.6	1.1

^*a*^Total RNA was isolated from bacterial cells recovered from infected plant tissues. Transcript abundance of *acrA* and *acrD* was determined by quantitative RT-PCR and was compared to RT-PCR signal from cells grown in LB broth to an OD_600_ of 0.5.

^*b*^Bacteria were re-isolated from infected shoots of apple rootstock 1, 4 and 7 days post inoculation (dpi).

^*c*^Bacteria were re-isolated from infected immature pears 12 hours post inoculation (hpi).

For apple rootstock infections, bacteria were re-isolated 1, 4 and 7 days after inoculation, respectively, and compared the abundance of *acrA* and *acrD* transcripts with cells grown in LB broth (Table 3). Due to the high activity of the *acrA* promoter in LB broth, expression analysis by quantitative RT-PCR revealed a down regulation of this gene *in planta*. On the other hand, since *acrD* was only expressed at a low level during cellular growth in LB broth, it showed a more than 3-fold induction *in planta*.

### Regulation of the RND-type multidrug efflux pump AcrD in *E. amylovora*

In other enterobacteria, e.g., *E. coli* and *S. enterica*, BaeR is involved in the regulation of the RND-type efflux pumps MdtABC and AcrD [19,34]. BaeR is the response regulator of the two-component system BaeSR, which controls a small set of adaptive factors involved in a unique envelope stress response in *E. coli*[23]. A BLASTP search using the amino acid sequence of BaeR from *E. coli* K12 as the query identified a homologous sequence in the genome of *E. amylovora* CFBP1430 (GenBank:EAMY_2266). These homologues share 74% amino acid sequence identity with each other.

In order to test whether BaeR plays a role in the regulation of the *acrD* promoter in *E. amylovora*, we analyzed whether the published BaeR-binding site sequence motif from *E. coli* (5′-TTTTTCTCCATDATTGGC-3′) is present in the plant pathogen [35]. Indeed we identified a similar motif resembling the BaeR binding box located at position -166 to -148 bp upstream of the coding sequence of *acrD* in Ea1189: 5′-TT**C**TTC**A**C**G**AT**T**A**C**TGGC-3′ (bold letters indicate mismatches to the consensus sequence of *E. coli*).

**Figure 4:**
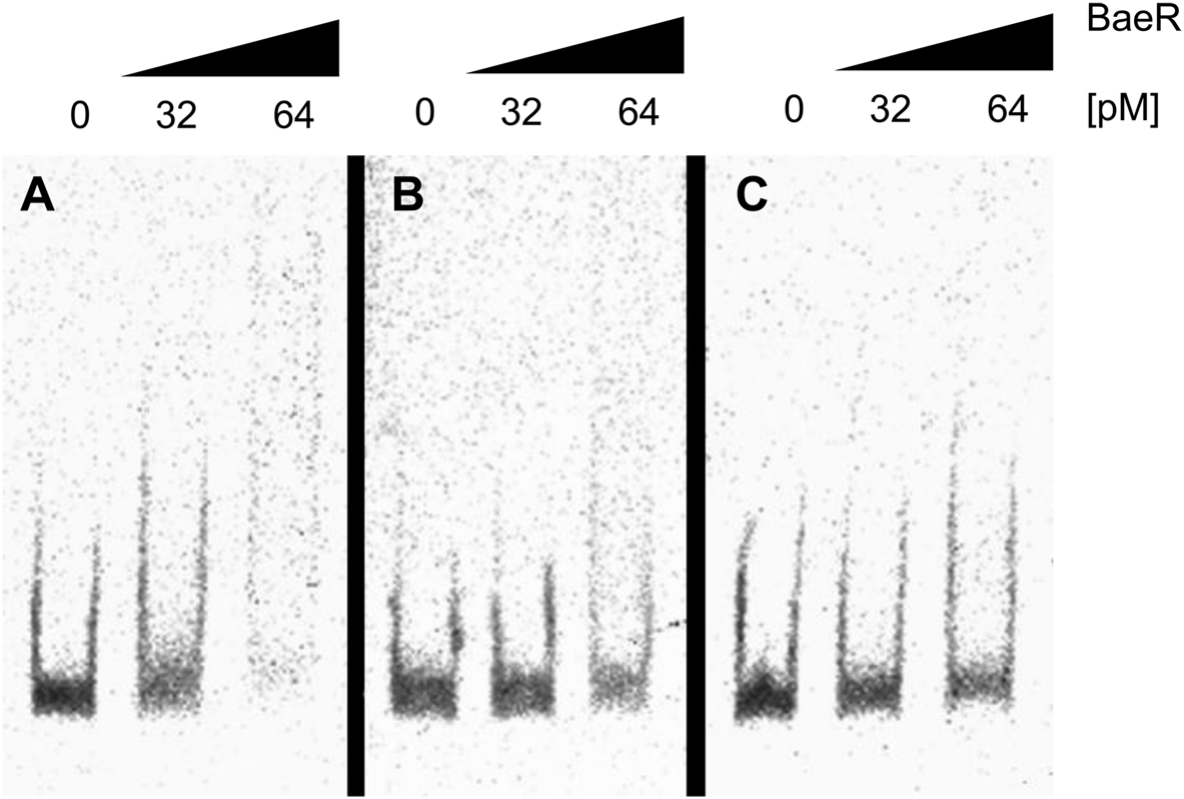
**Electrophoretic mobility shift analysis of BaeR interaction with Cy5-labeled DNA fragments.** DNA fragments contain the promoter regions of **(A)***acrD* (246 bp), **(B)***acrA* (205 bp) and **(C)***tolC* (291 bp), respectively. Approximately 0.16 pmol of the DNA fragments were incubated with increasing amounts of His-tag purified BaeR (indicated at the top of each lane). The DNA-protein complexes were separated on 4% non-denaturing polyacrylamide gels.

To confirm the binding of BaeR to the *acrD* promoter *in vitro*, an electrophoretic mobility shift assay (EMSA) was performed. DNA fragments used in the EMSA were the Cy5-labeled upstream region of *acrD* (246 bp), and as controls, the upstream regions of *acrAB* (205 bp) and *tolC* (291 bp). The DNA fragments were incubated with increasing amounts of purified BaeR protein in presence of nonspecific competitor DNA (Salmon sperm) (Figure 4). The purified BaeR protein showed binding to the upstream region of *acrD* with increasing concentrations, which was detected as a smear (Figure 4A). A slight interaction between the *acrAB* promoter and BaeR was detected at the highest protein concentration (64 pM), which could suggest an unspecific binding (Figure 4B). No interactions between the fragment of the promoter region of *tolC* and BaeR were detected (Figure 4C). These results show that BaeR binds to the *acrD* regulatory region and is probably involved in its regulation.

### Induction of *acrD* through overexpression of BaeR

Owing to the interaction between the *acrD* promoter region and BaeR observed during EMSA assays, we investigated whether overexpression of BaeR may induce the expression of *acrD*. Therefore, we cloned *baeR* under an arabinose-inducible promoter (pBAD24.baeR) and performed qRT-PCR analysis 1 hour after induction. Relative fold-changes in mRNA transcripts of *acrA* (0.8-fold), *acrD* (3.8-fold) and *tolC* (0.7-fold) were determined. The obtained data values correlate with the observed interaction of BaeR during EMSA indicating a specific binding of BaeR to the regulatory region of *acrD*.

## Discussion

Bacteria have evolved energy-dependent multidrug efflux pumps in order to prevent intracellular accumulation of toxic compounds, including antimicrobials, antibiotics, dyes and detergents [6,34]. In several enterobacteria, including the human pathogen *E. coli* and the plant pathogen *E. amylovora*, the RND transporter AcrAB-TolC has been described as the major multidrug efflux system providing resistance towards lipophilic and amphiphilic substrates but not towards hydrophilic compounds [6,16]. Another member of the RND family, AcrD, has been shown to efficiently efflux highly hydrophilic aminoglycosides from *E. coli* cells [13]*.* Here, we identified a gene encoding AcrD in *E. amylovora* Ea1189, which shows significant sequence homology to the cognate aminoglycoside efflux pump of *E. coli* and investigated the role of this transporter in the fire blight pathogen.

Due to the high level of homology shared by AcrD from *E. coli* and *E. amylovora*, it was not surprising to find similar substrate specificities. Previous studies of AcrD in *E. coli* have characterized it as an efflux transporter which provides resistance to hydrophilic aminoglycosides as well as to some amphiphilic compounds such as bile acids, novobiocin and fusidic acid [14,33]. It has also been reported that deletion of *acrD* does not cause hypersusceptibility to amphiphilic drugs [36,37], which may be due to low expression levels during cellular growth [14]. We have been able to detect similar low expression levels of *acrD* in *E. amylovora* Ea1189 during growth in LB broth (Figure 1A). Moreover, we were unable to detect hypersusceptibility to any of the tested antimicrobial compounds in an *acrD*-deficient mutant (Table 1).

As noted for other bacteria, the overproduction of AcrD in an *acrB*-deficient host led to increased resistance towards detergents, novobiocin and fusidic acid [14,35]. Overproduction of AcrD in an *acrB*-deficient mutant of *E. amylovora* Ea1189 increased the resistance to several antimicrobial compounds and heavy metals. It is noteworthy that expression of *acrD* under control of the *lac* promoter displayed only a minor effect on the resistance level compared to *acrD* expression driven by a combination of the *lac* promoter and the native promoter (up to 16-fold changes in MICs, Table 1). It has previously been reported that strong overproduction of AcrD may interfere with normal activity of the pump [14].

In this study, we identified two new substrates, clotrimazole and luteolin, which increased the substrate spectrum of AcrD in enterobacteria. Clotrimazole is a derivative of imidazole, commonly used in the treatment of fungal infections, and acts primarily by inhibiting the activity of cytochrome P450 mono-oxygenase [38]. Luteolin is one of the most common flavonoids present in many plant families. One of the functions of flavonoids in plants is their protective role against microbial invasion. Luteolin was shown to inhibit bacterial N-acetyltransferase activity [39].

Since AcrD conferred resistance to aminoglycosides in *E. coli*[13], we hypothesized that AcrD of *E. amylovora* would display a similar substrate spectrum. However, overexpression of AcrD in *E. amylovora* Ea1189-3 did not increase the MICs of the aminoglycosides amikacin, gentamicin, streptomycin, and tobramycin. Although it is important to note that we observed occasional, but not reproducible, 2-fold differences between the aminoglycoside MICs for different experiments (data not shown). While this result is contradictory to previous findings for *E. coli*[13], it may reflect a possible adaptation of the AcrD transporter to a particular physiological function during growth in the plant environment.

To elucidate the role of AcrD in the plant environment, we analyzed whether this RND-type efflux pump is involved in pathogenesis of the plant pathogen. Previously, we have observed that disruption of the AcrB efflux pump in *E. amylovora* significantly reduced virulence on apple rootstock [16]. This prompted us to evaluate the effect of AcrD on the virulence of the fire blight pathogen by studying development of disease symptoms. However, one week after inoculation, the wild type as well as the *acrD*-deficient mutant conferred typical symptoms on all inoculated shoots, indicating that the disruption of the efflux pump had negligible impact on virulence on apple rootstock. As the host range of *E. amylovora* also includes pear trees, we further investigated the virulence of the wild type and its *acrD*-deficient mutant on immature pear fruits (cv. ‘Bartlet’) with the conclusion that AcrD is not involved in the interaction of the fire blight pathogen with this host.

Additionally, we studied the expression levels of the AcrAB and AcrD efflux pumps *in vitro* and *in planta*, respectively. The activity of the *acrA* promoter was lower *in planta* than in LB medium (Table 3). However, it is possible that growth of the bacteria in LB broth may increase expression of the AcrAB pump. A similar induction of the RND-type efflux system MexAB-OprM in *Pseudomonas syringae* was observed during growth in complex King’s B medium [40]. Specific components of the complex media might induce the expression of these RND efflux systems. Alternatively, the efflux pumps may play a role in the secretion of metabolites during exponential growth of bacteria in complex medium.

The level of *acrD* expression was low during growth in LB medium (Figure 1B), whereas it was slightly induced *in planta* (Table 3) indicating that plant-derived compounds are able to induce the AcrD pump. The nature of these compounds remains to be elucidated.

Several multidrug transporters are induced in response to the presence of toxic substances [18]. We identified the substrates deoxycholate, naringenin, tetracycline, novobiocin, fusidic acid, tannin and zinc as inducers of *acrD* in *E. amylovora*. In prokaryotes, the expression of drug transporter genes is frequently mediated by transcriptional regulatory proteins, whose genes are often located adjacent to those encoding the transport system. However, no local transcriptional regulator was identified flanking the *acrD* gene in *E. amylovora*, suggesting that expression of *acrD* may be subject to regulation at the global level.

The *acrD* gene belongs to the regulon of the envelope stress response, two-component system BaeSR in *E. coli* and *Salmonella enterica*. A *baeSR*-deficient mutant of *E. amylovora* Ea1189 has previously been evaluated for virulence on immature pears, and exhibit full-virulence, as that of wild type, on immature pear fruits [41]. The core regulon of BaeSR consists of *spy*, encoding a protein chaperon, and the RND efflux pump genes *acrD* and *mdtABC*[42]. Interestingly, we identified a partial overlap between the compounds inducing expression of *acrD* in *E. amylovora* and *baeR* in *E. coli*, e.g., flavonoids (naringenin), zinc, and tannin [24,42]. Accordingly, the contribution of the two-component system BaeSR to regulation of the *acrD* gene in *E. amylovora* became of particular interest to us. In *E. coli* and *S. enterica*, BaeR, upon activation by phosphorylation through BaeS, binds to the upstream promoter region of *mdtA* and *acrD*[19,35]. Our results showed that BaeR of *E. amylovora* is able to bind the promoter region of *acrD* in *E. amylovora*, but not to the promoter regions of *acrA* or *tolC* (Figure 4). Additional investigation of the regulatory networks controlling expression of *acrD* in growth cultures and in natural environments, such as within host plants, will need to be conducted in order to provide further insights into the role of this multidrug transporter in the physiology of the cell.

In summary, we have identified a homologue of the RND-type multidrug efflux pump AcrD in *E. amylovora* Ea1189. Despite the fact that AcrD of Ea1189 was unable to efflux aminoglycosides, we detected a similar substrate spectrum compared to homologues of AcrD from other enterobacteria. Finally, we identified two substrates, clotrimazole and luteolin, hitherto unreported as substrates of AcrD in *E. coli* and *S. enterica*.

## Conclusions

The aim of the present study was the characterization of AcrD, a RND-type multidrug efflux pump from the plant pathogen *E. amylovora,* causing fire blight on apple and pear*.* Our results demonstrated that AcrD plays a role in drug resistance to a limited number of amphiphilic compounds. We showed that the substrate specificity of AcrD from *E. amylovora* and of AcrD from *E. coli* is partly overlapping. However, in contrast to AcrD from *E. coli*, AcrD from *E. amylovora* cannot provide resistance towards aminoglycosides. The expression of *acrD* was up-regulated by the addition of several substrates and was found to be regulated by the envelope stress two-component regulatory system BaeSR. An *acrD* mutant showed full virulence on apple rootstock and immature pear fruits.

## Methods

### Bacterial strains, plasmids and growth conditions

Bacterial strains and plasmids used in this study are listed in Table 4. *E. amylovora* strains were cultured at 28°C in Lysogeny Broth (LB) or on LB plates. *E. coli* XL-1 Blue was used as cloning host. *E. coli* cells were routinely maintained at 37°C in LB or double Yeast Trypton (dYT) medium. Cultures harboring individual vectors were supplemented with 50 μg/ml ampicillin (Ap) for *E. coli* or 250 μg/ml for *E. amylovora*, 25 μg/ml chloramphenicol (Cm), 2 μg/ml gentamicin (Gm) and 25 μg/ml kanamycin (Km) when necessary. Bacterial growth was monitored using a spectrophotometer at 600 nm (OD_600_).

**Table 4:** Bacterial strains and plasmids used in this study

***Plasmid or strain***	***Relevant characteristics or genotype***^**a**^	***Reference or source***
**Plasmid**		
pJET1.2	Ap^r^, rep (pMB1) from pMBI responsible for replication	Thermo scientific
pCAM-MCS	Ap^r^, pCAM140-derivative without mini-Tn*5*, contains the MCS of pBluescript II SK (+)	[16]
pFCm1	Ap^r^, Cm^r^, source of Cm^r^ cassette flanked by FRT sequences	[43]
pCAM-Km	Km^r^, variant of the gene replacement vector pCAM-MCS, Ap^r^ replaced by Km^r^	This study
pCAM-Km.acrD-Cm	Km^r^, Cm^r^, contains a 1.1-kb fragment of *acrD* from *E. amylovora* Ea1189, insertion of 1153-bp Cm-FRT cassette from pFCm1 in BamHI site	This study
pBlueScript II SK(+)	Ap^r^, ColE1 origin	Stratagene
pBlueScript II KS(+)	Ap^r^, ColE1 origin	Stratagene
pBBR1MCS	Cm^r^, ColE1 origin	[44]
pBlueKS.acrD	Ap^r^, contains a 3.1-kb fragment carrying *acrD* of *E. amylovora* Ea1189 under control of *lac* promoter	This study
pBlueKS.acrD-ext	Ap^r^, contains a 3.5-kb fragment carrying *acrD* of *E. amylovora* Ea1189 including promoter region under control of *lac* promoter	This study
pBlueSK.acrD	Ap^r^, contains a 3.1-kb fragment carrying *acrD* of *E. amylovora* Ea1189 in opposite orientation with respect to *lac* promoter	This study
pBlueSK.acrD-ext	Ap^r^, contains a 3.5-kb fragment carrying *acrD* of *E. amylovora* Ea1189 including promoter region in opposite orientation with respect to *lac* promoter	This study
pBBR.egfp.TIR	Cm^r^, contains the TIR-*egfp*-T_0_ cassette in pBBR1MCS in opposite orientation with respect to *lac* promoter	[16]
pBBR.acrD-Pro.egfp	Cm^r^, contains a 206-bp fragment carrying the promoter region of *acrD*, transcriptional fusion of *acrD* with *egfp*	This study
pBBR.acrA-Pro.egfp	Cm^r^, contains a 133-bp fragment carrying the promoter region of *acrA*, transcriptional fusion of *acrA* with *egfp*	This study
pBlueSK.baeR	Ap^r^, contains a 0.7-kb fragment carrying *baeR* of *E. amylovora* Ea1189 under control of *lac* promoter	This study
pET-28a(+)	Km^r^, f1 origin	Novagen
pET28a.baeR	Km^r^, contains a 0.7-kb fragment carrying *baeR* of *E. amylovora* Ea1189, C-terminal translational fusion with His-tag	This study
pCP20	Cm^r^, Ap^r^, contains yeast Flp recombinase gene, rep (pSC101) responsible for temperature-sensitive replication	[45]
pBAD24	Ap^r^, pMB1 origin, *araC*	[46]
pBAD24.baeR	Ap^r^, contains a 0.7-kb fragment carrying *baeR* of *E. amylovora* Ea1189 under control of P_BAD_ promoter	This study
**Strain**		
*Escherichia coli*		
XL1-Blue	endA1 gyrA96(nal^R^) thi-1 recA1 relA1 lac glnV44 F'[ ::Tn10 proAB^+^ lacI^q^ Δ(lacZ)M15] hsdR17(r_K_^-^ m_K_^+^)	Stratagene
TG1	K-12 supE thi-1 Δ(lac-proAB) Δ(mcrB-hsdSM)5, (r_K_^-^m_K_^-^)	[47]
KAM3	*acrAB* mutant of TG1	[48]
BL21(DE3)	F^–^ ompT gal dcm lon hsdS_B_(r_B_^-^ m_B_^-^) (λDE3)	Novagen
S17-1	TpR SmR recA, thi, pro, hsdR-M^+^RP4: 2-Tc:Mu: Km	[49]
S17-1 λ-pir	λpir phage lysogen of S17-1	[49]
DH5α λ-pir	sup E44, ΔlacU169 (ΦlacZΔM15), recA1, endA1, hsdR17, thi-1, gyrA96, relA1, λpir phage lysogen	D. Lies, Caltech
*Erwinia amylovora*		
Ea1189	Wild type	GSPB^*b*^
Ea1189-3	Km^r^, *acrB* mutant carrying Km^r^ cassette in the *acrB* gene	[16]
Ea1189.acrD	*acrD* mutant	This study

^*a*^Ap^r^, ampicillin resistance; Cm^r^, chloramphenicol resistance; Km^r^, kanamycin resistance.

^*b*^GSPB, Göttinger Sammlung Phytopathogener Bakterien, Göttingen, Germany.

### PCR amplifications, modifications and protein purification

Primers (see Additional file 6) were designed based on *E. amylovora* CFBP1430 genome sequences available from NCBI (GenBank NC_013961.1). Screening PCR reactions were carried out using the DreamTaq DNA polymerase (Thermo Scientific) in accordance with the manufacturer’s instructions and optimized annealing temperatures based on the melting temperatures of the respective primers. For high fidelity PCR reactions, Phusion DNA polymerase (Thermo Scientific) was used where the annealing temperature was 3°C higher than the lower temperature of the used primer combination.

Restriction enzyme (Thermo Scientific) and T4 DNA ligase (Thermo Scientific) reactions were performed as per the manufacturer’s instructions at the appropriate temperature where all ligation reactions were incubated at room temperature.

DNA purifications were either performed using the GeneJET PCR purification kit (Thermo Scientific) or the GeneJET Gel extraction kit (Thermo Scientific) following the manufacturer’s instructions.

Protein purification was carried out using the Ni-NTA Spin Kit (Qiagen) following the manufacturer’s instructions.

### Construction of the *E. amylovora acrD*-deficient mutant

A 1058-bp fragment located in the *acrD* gene was amplified using the primer pair acrD_ko_fwd and acrD_ko_rev and verified by sequencing. A chloramphenicol cassette flanked by Flp-*FRT* sites was cut from plasmid pFCM1 and inserted into *Bam*HI-digested pJET.acrD-ko, yielding pJET.acrD-ko.Cm. A 2.2-kb *Eco*RI fragment cut from pJET.acrD-ko.Cm was ligated into *Eco*RI-digested pCAM-Km, yielding the final replacement plasmid pCAM-Km.acrD-Cm. The plasmid was transformed into electrocompetent cells of *E. amylovora* Ea1189, which subsequently were grown for 3 h at 28°C in dYT broth. Putative mutants were screened for homologous recombination events by testing their antibiotic resistance. Mutants that resulted from single crossover events were identified by their ability to grow on plates containing Km. In order to confirm gene disruption through a double crossover event in Cm-resistant and Km-sensitive colonies, primers acrD_fwd and acrD_rev were designed, which bind upstream and downstream, respectively, of the 1058-bp *acrD* fragment used for generation of the gene replacement vector. PCRs were done using these locus-specific primers with primers binding in the Cm cassette (cat_out2, cat_out3, cat_out4, cat_out5). Amplified PCR products were verified by sequencing.

The Cm-*FRT* cassette was finally excised using the temperature-sensitive plasmid pCP20 that carries the yeast Flp recombinase gene [43,45]. Briefly, Cm-resistant mutants of Ea1189 were transformed with pCP20 and selected at 28°C on LB plates containing Ap. Subsequently, Ap-resistant transformants were streaked on non-selective agar plates and incubated at 43°C for 1 h; following incubation at 28°C for 48–60 h. Single colonies were selected and tested on agar plates containing Cm or Ap to confirm successful excision of the Cm cassette and loss of plasmid pCP20.

### Construction of *acrD* overexpression plasmids

A 3.06-kb fragment containing *acrD* was amplified from *E. amylovora* Ea1189 using the primer pair acrD-ApaI and acrD-SacI. The PCR product was sequenced and further cloned into *Apa*I-*Sac*I-digested pBlueScript II KS(+) and pBlueScript II SK(+), respectively (pBlueKS.acrD, pBlueSK.acrD).

Next, a 210-bp fragment containing the upstream region of *acrD* was amplified using the primers narP-ApaI and acrD_SalI. The PCR product was cloned into a *Sal*I restriction site located in the beginning of the *acrD* gene (pBlueKS.acrD-ext, pBlueSK.acrD-ext).

### Drug susceptibility tests

The minimal inhibitory concentrations (MIC) of drugs for *E. amylovora* strains were determined by a 2-fold dilution assay in Mueller-Hinton broth (MHB). All tests were done in at least triplicate following the Clinical and Laboratory Standards Institute recommendations [50]. Growth of bacteria at 28°C was examined by visual inspection after 48 h incubation. The MIC was defined as the lowest concentration of an antibiotic that completely prevented visible cell growth.

### Generation of promoter-EGFP fusions

Transcriptional fusions between the promoter regions of *acrA* and *acrD*, respectively, and *egfp* were created using a previously described PCR-based method [51]. Briefly, a 546-bp fragment containing the upstream region of *acrD* was amplified using the primer acrD_up and the reverse primer acrD-P-egfp containing a 24-nt extension that is homologous to the start of the *egfp* gene. The *acrA* upstream region was amplified using the primer acrAB_fwd and the reverse primer acrA-P-egfp. Next, the reporter gene *egfp* was amplified using the primer pair egfp-ATG and egfp-Cm and the plasmid pBBR.egfp.TIR [16] as the template. All PCR products were gel-purified. For the fusion reaction, 200 ng of a PCR fragment containing a promoter region were mixed with 200 ng of the reporter gene fragment. Nested primer pairs were used for the fusion PCR reactions. For fusion of the *acrD* promoter to the *egfp* gene, the primers acrD-P-fwd_SacII-2 and uidA-t0-KpnI were used. The primers acrA-P-fwd-SacII and uidA-t0-KpnI were used in a PCR to fuse the *acrA* promoter to *egfp*. The PCR products were gel-purified to remove non-fused fragments. Next, the fusion product was cloned in opposite direction to the *lacZ’* promoter, into *Sac*II-*Kpn*I-treated pBBR1MCS, yielding plasmids pBBR.acrA-Pro.egfp and pBBR.acrD-Pro.egfp.

### Promoter activity of *acrD in vitro*

The reporter gene *egfp* was employed to study the impact of diverse antimicrobial substances on promoter activities of *acrD* in *E. amylovora*. Plasmids carrying the transcriptional fusions were transformed into Ea1189. Antimicrobial compounds were added to the bacterial cells in 96-well microtiter plates by the 2-fold dilution method as described for MIC assays. EGFP fluorescence of the cells following exposure to various concentrations of the substrates was measured 48 hours after incubation at 28°C using the microplate reader Infinite M1000 PRO (Tecan, Crailsheim, Germany) with an excitation wavelength of 470 nm and emission detection at 516 nm.

Fluorescence values obtained were plotted versus optical density in a scatter plot (see Additional file 5). A best-fit linear regression line was added to the plot and a 95% confidence interval determined. Data points that did not meet the confidence interval criteria indicate fluorescence values higher than the average, thereby suggesting induction of the *acrD* promoter by the respective compound.

To further demonstrate promoter induction, the identified substrates were tested in liquid cultures. Cells of Ea1189 harboring plasmid pBBR.acrD-Pro.egfp were incubated in LB broth supplemented with each substrate for 24 hours, then harvested by centrifugation, resuspended in phosphate-buffered saline, adjusted to an OD_600_ value of 0.1 and fluorescence determined.

### Apple plant material and inoculation procedures

Apple plants (rootstock Malus MM106) were grown in a greenhouse at 20 to 25°C, 60% humidity, and 12 h photoperiod (15,000 lx). *E. amylovora* Ea1189 and its *acrD* mutant, grown on LB agar for 24 h, were resuspended and diluted to a cell density of 1 x 10^6^ CFU/ml in sterile demineralized water. Apple plants were inoculated by prick technique [52]. Each bacterial strain was inoculated into one shoot of five single plants. A bacterial suspension (5 μl) was placed onto each wound on the shoot tip. Plants were monitored for symptom development daily. Survival of bacteria in plant tissue was examined by re-isolation of bacterial cells 1 and 5 day(s) after inoculation, respectively, from 1 cm of the shoot tip around the inoculation area. Ultimately, five wounds were pooled together, homogenized in 0.9% NaCl, serially diluted, and spread on LB agar plates. The experiment was repeated in triplicate.

In order to analyze the abundance of *acrA* and *acrD* mRNA transcripts in *E. amylovora* Ea1189 during growth in apple rootstock MM106, total RNA was isolated from infected apple shoots 1, 4 and 7 day(s) post inoculation, respectively. Five individual wounds were pooled together, homogenized in 0.9% NaCl and centrifuged for 2 min at 4000 rpm. The supernatant was transferred to 15 ml killing buffer (20 mM Tris–HCl, pH 7.5; 20 mM NaN_3_) [53] and centrifuged for 20 min at 4000 rpm. The supernatant was decanted and the pellet frozen at -80°C for further RNA extraction.

### Virulence assay on immature pears

Virulence of *E. amylovora* Ea1189 and its *acrD* mutant was determined on immature pears (cv. ‘Bartlet’). Bacteria, grown at 28°C on LB agar plates for 24 h, were resuspended and adjusted to an OD_600_ of 1.0 in sterile demineralized water for inoculation. Immature pear fruits were surface-sterilized and pricked with a sterile needle as described previously [54]. Wounds were inoculated with 5 × 10^6^ CFU/ml and incubated in a humidified chamber at room temperature for 8 days. Disease symptoms were recorded by means of diameter of necrosis surrounding the infection site. Fruits were assayed in triplicates and the experiment was repeated twice.

To analyze gene expression of *E. amylovora* Ea1189 during growth on pear fruits, immature fruits were cut in slices (approx. 0.5 cm). Five slices were inoculated with 100 μl of a bacterial suspension adjusted to an OD_600_ of 1.0 in sterile demineralized water. The suspension was evenly distributed on the slice and incubated for 12 hours in a humidified chamber at room temperature. Next, the upper layer of the surface was scratched from the five slices, resuspended in 25 ml of PBS and centrifuged for 2 min at 4000 rpm. The supernatant was transferred to 15 ml killing buffer and further processed as described above.

### RNA isolation and quantitative real-time PCR

Cell cultures were grown in LB broth until the desired optical densities were achieved. An aliquot containing 15 × 10^9^ CFU (equivalent of 15 ml OD_600_ of 1.0) was transferred to 15 ml killing buffer and centrifuged for 20 min at 4000 rpm. The supernatant was decanted and the pellet frozen at -80°C for further RNA extraction.

Total RNA was isolated by acid phenol/chloroform extraction [53]. The obtained RNA was treated with DNAse (Ambion/Life Technologies, Darmstadt, Germany) and subsequently checked for purity by gel electrophoresis and determination of the A260/A280 and A260/A230 ratios using a Nanodrop ND-2000 spectrophotometer (Thermo Fischer Scientific). High quality RNA was reverse transcribed and amplified with the OneStep RT-PCR Kit according to the manufacturer’s protocol (Qiagen, Hilden, Germany). Template RNA (5 ng) was used in a standard 25-μl qRT-PCR reaction with specific primers (see Additional file 6). As negative control, RNA samples without reverse transcriptase were included to detect possible DNA contaminations.

For analysis, a Mastercycler ep *realplex*^2^ gradient S (Eppendorf, Hamburg, Germany) was used. Cycling parameters included a 15 min initial denaturation at 95°C to activate the DNA polymerase followed by 40 cycles consisting of 15 sec at 95°C, 30 sec at 55°C and 30 sec at 72°C. The final step consisted of 1 min at 95°C and 30 sec at 55°C. A melting curve analysis with a temperature ramp from 25°C to 95°C in 20 min was performed at the end of each run to determine specificity of amplified qPCR products.

Each sample was analyzed for gene expression in triplicate. Quantification of mRNA transcripts was performed by the comparative C_t_ method. Briefly, the C_t_ values of the samples of interest were compared with a non-treated sample. All C_t_ values were normalized to the housekeeping gene *recA*, which shows constant expression at different ODs and medium compositions as well as similar amplification efficiency to the target genes [55]. The comparative C_t_ method was calculated by $2^{-\Delta C_{t},\mathrm{sample}-\Delta C_{t},\mathrm{reference}}$, where ΔC_t_ was normalized to the endogenous housekeeping gene *recA*. Subsequently, fold-changes between the samples were determined based on the calculated C_t_ method.

### Expression of the BaeR protein

Expression of BaeR was achieved by using the vector pBAD24 where the expression is controlled by the P_BAD_ promoter and *araC*. Therefore, we cloned *baeR* under control of the arabinose inducible promoter (pBAD24.baeR) and transformed the plasmid into *E. amylovora* wild-type cells. Protein expression was induced by adding 1% L-arabinose when cultures reached an OD_600_ of 0.5. Cells were further incubated for 1 hour at 28°C and subsequently harvested by centrifugation.

### Electrophoretic mobility shift assay

DNA fragments used for the electrophoretic mobility shift assay (EMSA) were PCR amplified using Cy5-labeled primers to perform a non-radioactive EMSA. DNA fragments used were the upstream region of *acrD* (246 bp), and as controls, the upstream regions of *acrAB* (205 bp) and *tolC* (291 bp). Approximately 0.16 pmol of Cy5-labeled DNA was mixed with increasing concentrations of His-tagged BaeR protein in a binding buffer reaction (50 mM Tris–HCl, pH 7.5; 1 mM DTT; 500 mM MgCl_2_; 100 mM EDTA; 10 mM NaCl; 5% glycerol). To decrease unspecific binding, 500 ng competitor DNA (Salmon sperm DNA, AppliChem) was added to the reaction. Incubation was done at room temperature for 30 min. The total reaction was run on a native 4% polyacrylamide gel in 0.5x Tris-borate-EDTA (TBE) buffer at constant 25 mA. After electrophoresis, fluorescence signals of the labeled DNA were visualized using a FLA-3000 phosphorimager (Raytest, Straubenhardt, Germany).

### Statistical analysis

Statistical analysis was performed using R [56]. Differences between two groups were determined by a two-sided t-test with equal variances and were considered significant at P < 0.05. When necessary the standard deviation is presented in the graph when the average of several values was applied.

## Competing interests

The authors declared that they have no competing interests.

## Authors’ contributions

DP carried out the molecular work, participated in the bioinformatical analysis and drafted the manuscript. HW conceived of the study, participated in its design and coordination and helped to draft the manuscript. All authors read and approved the final manuscript.

## Supplementary Material

Additional file 1 **Phylogenetic tree of AcrD.** Description: The tree was calculated based on AcrD from *Erwinia amylovora* Ea1189 (black arrow) and its homologues from other members of the *Enterobacteriaceae* family, including *Erwinia pyrifoliae* (95% identity), *E. tasmaniensis* (93% identity), *E. billingiae* (83% identity), *Pantoea agglomerans* (82% identity), *P. ananatis* (79% identity), *Enterobacter cloacae* (79% identity), *Salmonella enterica* (79% identity), *Citrobacter koseri* (79%), *Klebsiella pneumoniae* (79% identity), *Escherichia coli* (78% identity) and *Shigella flexneri* (78% identity). The dendrogram was generated based on percentage of identity between the sequences using the neighbor joining algorithm implemented in Jalview [25-28]. Click here for file

Additional file 2 **Sequence alignment of AcrD from *Erwinia amylovora *** **Ea1189 and *Escherichia coli *** **K-12.** Description: The alignment is based on the amino acid sequences of AcrD using ClustalW for analysis and Jalview for data presentation. AcrD of Ea1189 is 79% identical and 89% similar to AcrD of *E. coli* K-12. Identical amino acid residues are shown in blue. Yellow bars show a quantitative measurement of conserved physico-chemical properties where the highest score shows amino acids of the same physico-chemical class [26-28]. Black bars indicate predicted transmembrane-spanning helices of AcrD from *E. amylovora*[29]. Click here for file

Additional file 3 **Modified view of the genomic organization of the *acrD *** **locus.** Description: The locus includes the region from *Erwinia amylovora* CFBP1430, *Escherichia coli* K-12 and *Salmonella enterica* serovar Typhimurium str. LT2, respectively, and was visualized by the Artemis Comparison Tool [57]. The gray areas indicate homologous regions with a minimum identity cutoff score of 88%. The region encoding *acrD* is highlighted in light gray. The alignment was performed using the nucleotide search BLASTN from NCBI. Click here for file

Additional file 4 **Membrane protein topology of AcrD from *Escherichia coli *** **K-12 (A) and *Erwinia amylovora *** **Ea1189 (B).** Description: The upper line indicates the predicted topology from TOPCONS [29] based on amino acid sequences. Red lines indicate an inner membrane orientation; blue lines indicate an outer membrane orientation. Grey boxes indicate transmembrane helices spanning from the inside to the outside, white boxes indicate transmembrane helices spanning from the outside to the inside. Below the line is a graphical interpretation of the reliability of the prediction for each amino acid. Click here for file

Additional file 5 **Scatter plot of the promoter activity of *acrD *** **from *E. amylovora *** **Ea1189.** Description: It shows the effect of substrates on the promoter activity of *acrD* as determined by a transcriptional fusion with the reporter gene e*gfp*. Antimicrobial compounds were added to cells of Ea1189 harboring pBBR.acrD-Pro.egfp by the 2-fold dilution method as described for MIC assays. EGFP fluorescence of the cells following exposure to various concentrations of the substrates was determined after 24 h incubation. A best-fit linear regression line between fluorescence and optical density values (dashed line) as well as a 95% confidence interval (solid line) are indicated. Outliers (black spots), showing higher fluorescence than the confidence interval, were identified as follows: deoxycholate (Doc), naringenin (Ng), tetracycline (Tc), and zinc sulfate (Zn). The following substrates were applied to this assay: (+)-catechin, acridine orange, acriflavine, amikacin, azithromycin, benzalkonium chloride, berberine, bile salts, cadmium acetate, chloramphenicol, ciprofloxacin, clarithromycin, clotrimazol, cobalt chloride, copper sulfate, crystal violet, deoxycholate, erythromycin, ethidium bromide, fusaric acid, fusidic acid, genistein, gentamycin, josamycin, luteolin, myricetin, naladixic acid, naringenin, nickel chloride, nitrofurantoin, norfloxacin, novobiocin, phloretin, polymyxin B, quercitin, rhodamine 6G, rifampicin, roxithromycin, SDS, silver nitrate, sodium arsenate, sodium tungstate, streptomycin, tetracycline, tetraphenylphosphonium chloride, tobramycin, and zinc sulfate. Click here for file

Additional file 6 Primers used in this study. Click here for file
