# Supplementary figures and images for: Characterization of AcrD, a Resistance-Nodulation-Cell Division-type multidrug efflux pump from the fire blight pathogen Erwinia amylovora
Source: BMC Microbiol. 2014 Jan 21;14:13. doi: 10.1186/1471-2180-14-13 (PMC3915751; doi:10.1186/1471-2180-14-13)

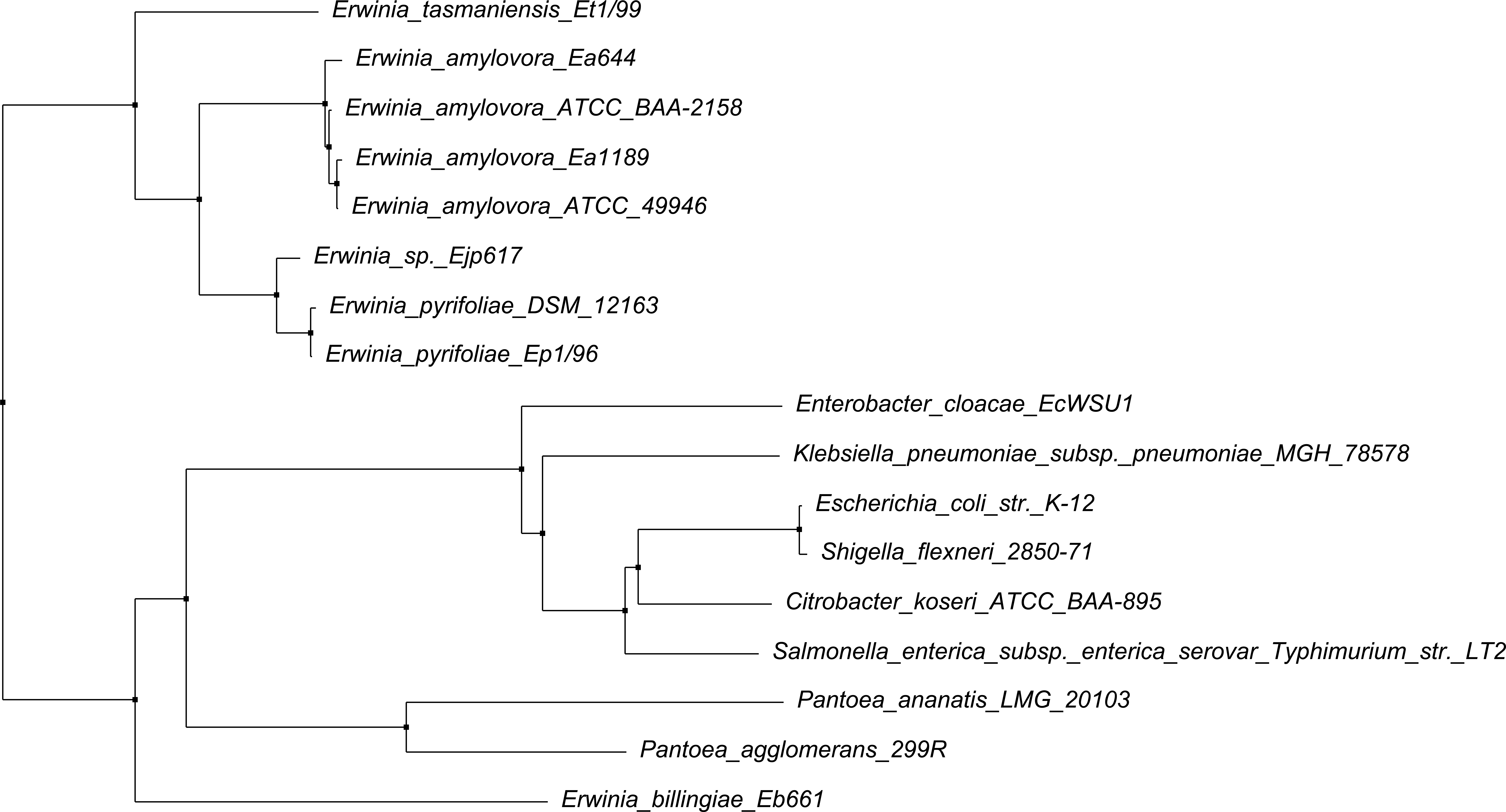

Supplement: Additional file 1 — Phylogenetic tree of AcrD. Description: The tree was calculated based on AcrD from Erwinia amylovora Ea1189 (black arrow) and its homologues from other members of the Enterobacteriaceae family, including Erwinia pyrifoliae (95% identity), E. tasmaniensis (93% identity), E. billingiae (83% identity), Pantoea agglomerans (82% identity), P. ananatis (79% identity), Enterobacter cloacae (79% identity), Salmonella enterica (79% identity), Citrobacter koseri (79%), Klebsiella pneumoniae (79% identity), Escherichia coli (78% identity) and Shigella flexneri (78% identity). The dendrogram was generated based on percentage of identity between the sequences using the neighbor joining algorithm implemented in Jalview [25-28]. [file 1471-2180-14-13-S1.tiff]

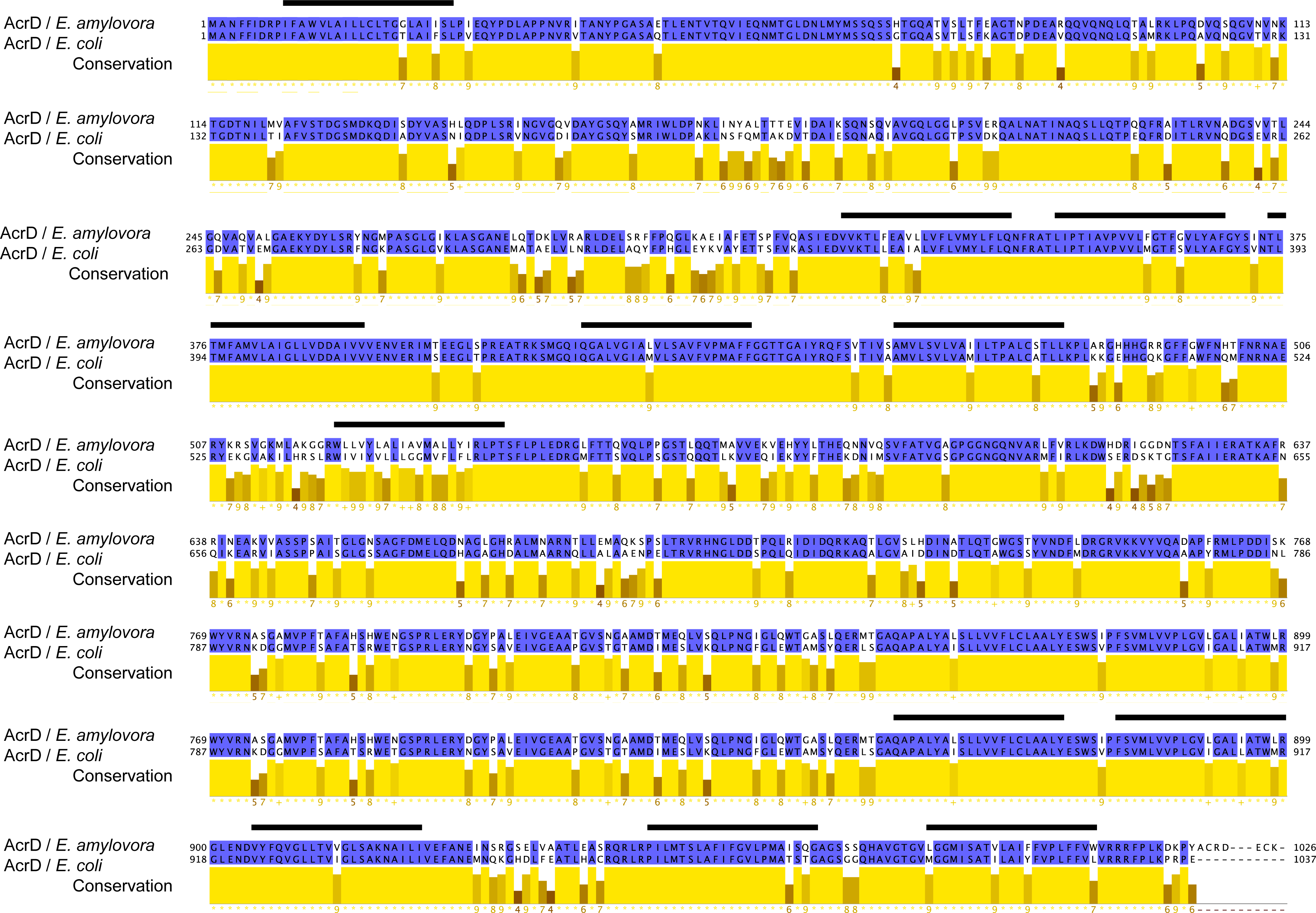

Supplement: Additional file 2 — Sequence alignment of AcrD from Erwinia amylovora Ea1189 and Escherichia coli K-12. Description: The alignment is based on the amino acid sequences of AcrD using ClustalW for analysis and Jalview for data presentation. AcrD of Ea1189 is 79% identical and 89% similar to AcrD of E. coli K-12. Identical amino acid residues are shown in blue. Yellow bars show a quantitative measurement of conserved physico-chemical properties where the highest score shows amino acids of the same physico-chemical class [26-28]. Black bars indicate predicted transmembrane-spanning helices of AcrD from E. amylovora[29]. [file 1471-2180-14-13-S2.tiff]

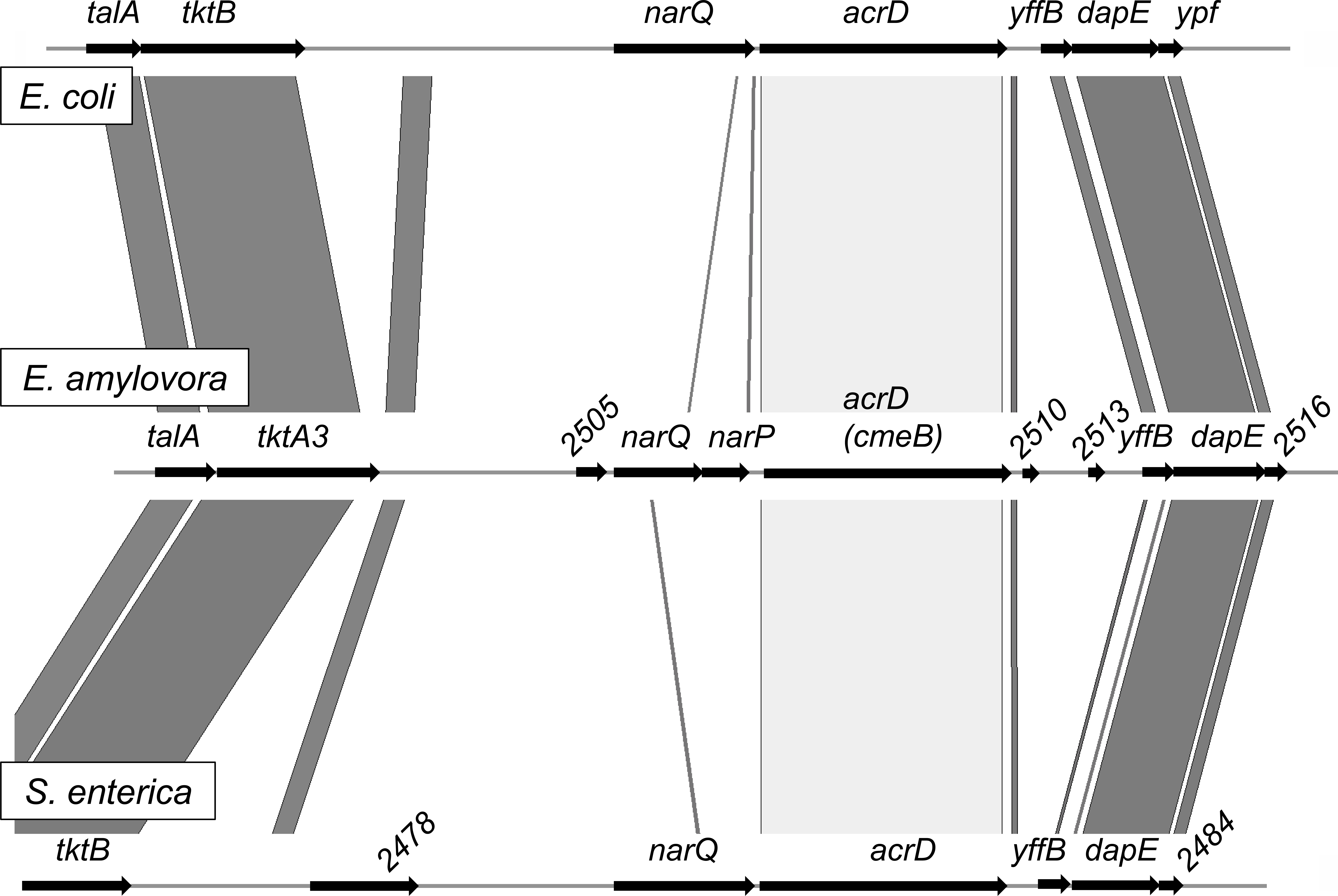

Supplement: Additional file 3 — Modified view of the genomic organization of the acrD locus. Description: The locus includes the region from Erwinia amylovora CFBP1430, Escherichia coli K-12 and Salmonella enterica serovar Typhimurium str. LT2, respectively, and was visualized by the Artemis Comparison Tool [57]. The gray areas indicate homologous regions with a minimum identity cutoff score of 88%. The region encoding acrD is highlighted in light gray. The alignment was performed using the nucleotide search BLASTN from NCBI. [file 1471-2180-14-13-S3.tiff]

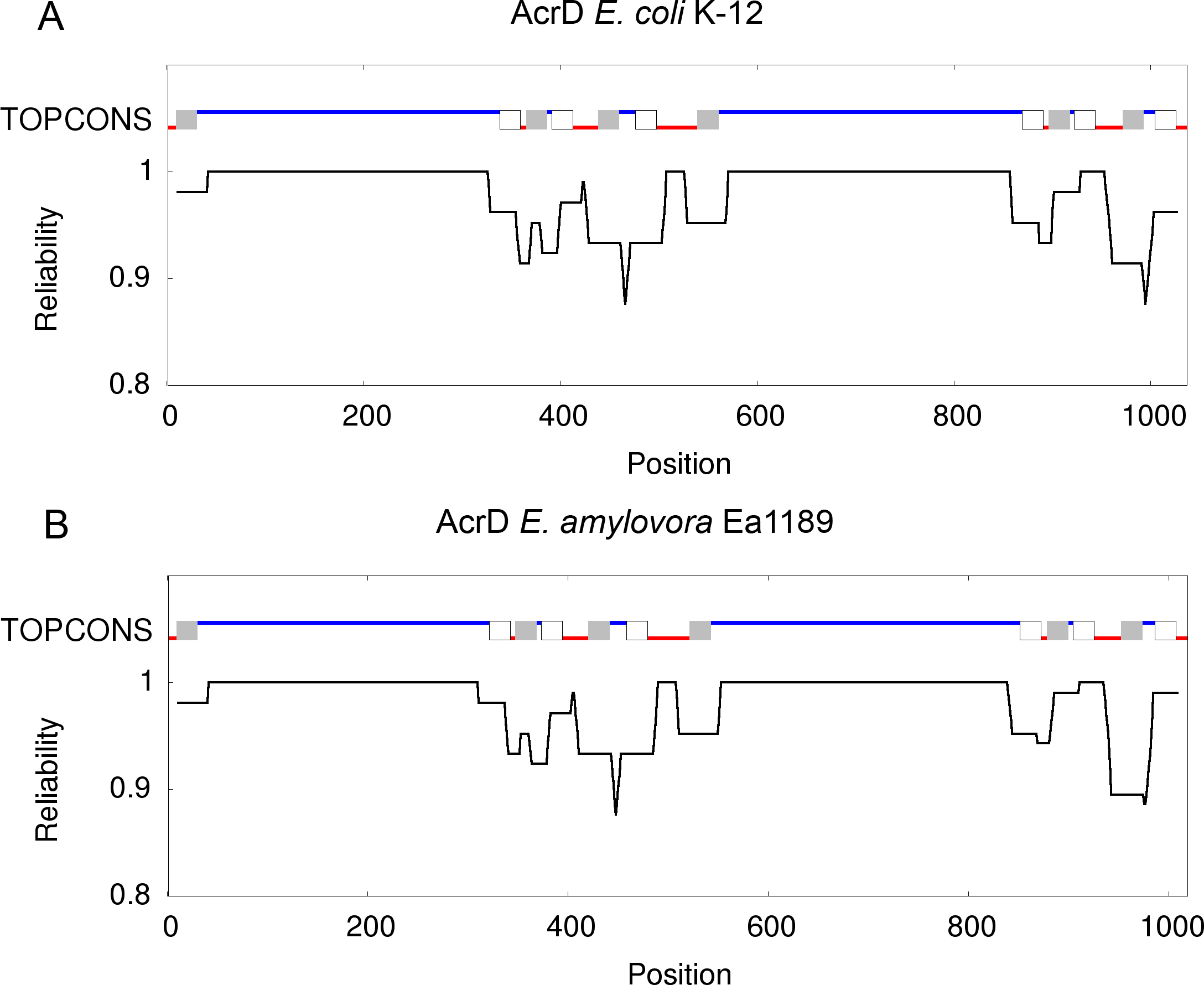

Supplement: Additional file 4 — Membrane protein topology of AcrD from Escherichia coli K-12 (A) and Erwinia amylovora Ea1189 (B). Description: The upper line indicates the predicted topology from TOPCONS [29] based on amino acid sequences. Red lines indicate an inner membrane orientation; blue lines indicate an outer membrane orientation. Grey boxes indicate transmembrane helices spanning from the inside to the outside, white boxes indicate transmembrane helices spanning from the outside to the inside. Below the line is a graphical interpretation of the reliability of the prediction for each amino acid. [file 1471-2180-14-13-S4.tiff]

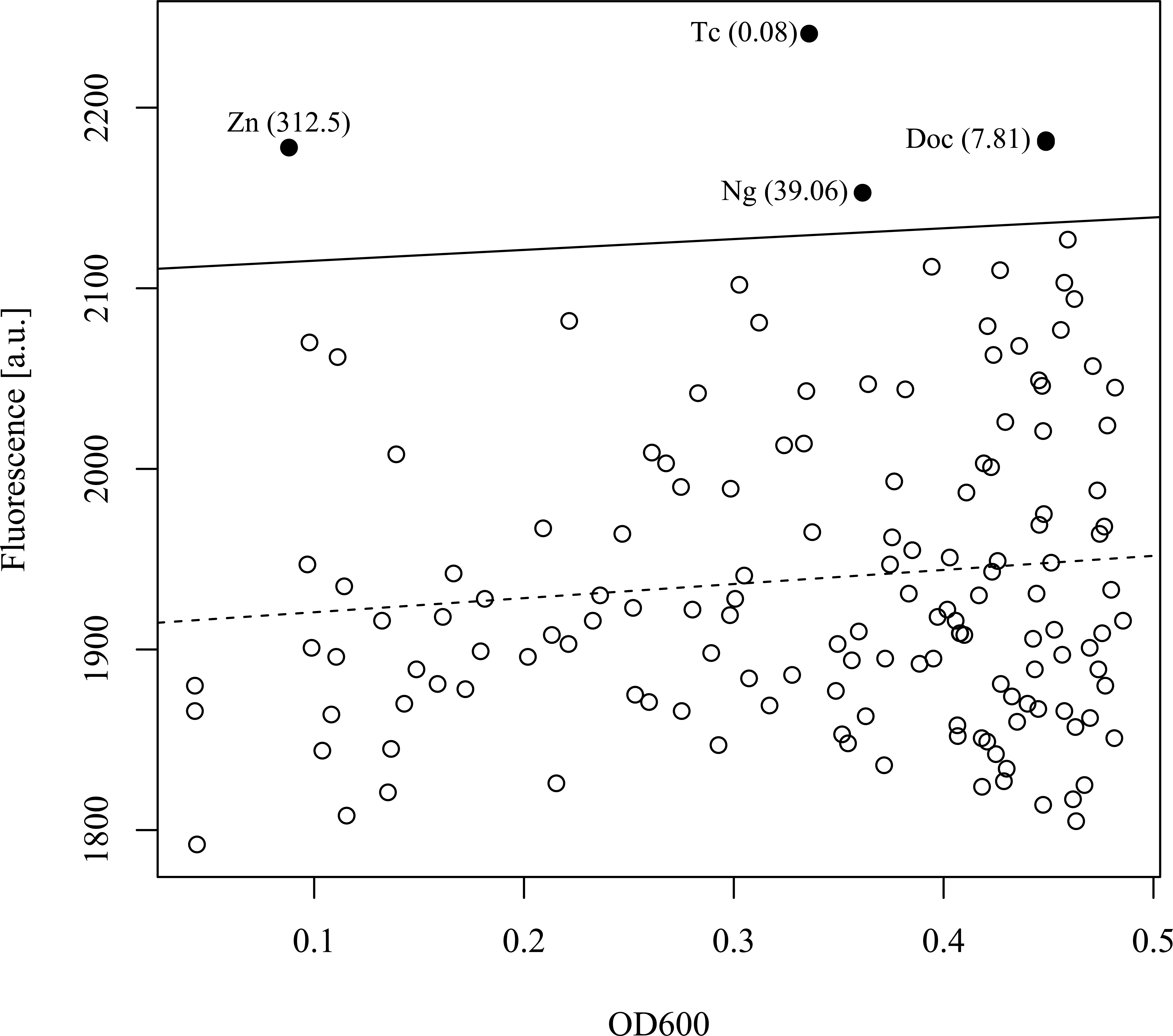

Supplement: Additional file 5 — Scatter plot of the promoter activity of acrD from E. amylovora Ea1189. Description: It shows the effect of substrates on the promoter activity of acrD as determined by a transcriptional fusion with the reporter gene egfp. Antimicrobial compounds were added to cells of Ea1189 harboring pBBR.acrD-Pro.egfp by the 2-fold dilution method as described for MIC assays. EGFP fluorescence of the cells following exposure to various concentrations of the substrates was determined after 24 h incubation. A best-fit linear regression line between fluorescence and optical density values (dashed line) as well as a 95% confidence interval (solid line) are indicated. Outliers (black spots), showing higher fluorescence than the confidence interval, were identified as follows: deoxycholate (Doc), naringenin (Ng), tetracycline (Tc), and zinc sulfate (Zn). The following substrates were applied to this assay: (+)-catechin, acridine orange, acriflavine, amikacin, azithromycin, benzalkonium chloride, berberine, bile salts, cadmium acetate, chloramphenicol, ciprofloxacin, clarithromycin, clotrimazol, cobalt chloride, copper sulfate, crystal violet, deoxycholate, erythromycin, ethidium bromide, fusaric acid, fusidic acid, genistein, gentamycin, josamycin, luteolin, myricetin, naladixic acid, naringenin, nickel chloride, nitrofurantoin, norfloxacin, novobiocin, phloretin, polymyxin B, quercitin, rhodamine 6G, rifampicin, roxithromycin, SDS, silver nitrate, sodium arsenate, sodium tungstate, streptomycin, tetracycline, tetraphenylphosphonium chloride, tobramycin, and zinc sulfate. [file 1471-2180-14-13-S5.tiff]
