# Supplementary material for: Characterization of AcrD, a Resistance-Nodulation-Cell Division-type multidrug efflux pump from the fire blight pathogen Erwinia amylovora
Source: BMC Microbiol. 2014 Jan 21;14:13. doi: 10.1186/1471-2180-14-13 (PMC3915751; doi:10.1186/1471-2180-14-13)
Supplement: Additional file 6 — Primers used in this study. [file 1471-2180-14-13-S6.docx]

**Supplementary TABLE 1** Primers used in this study

| *Primer* | *Sequence (5' - 3')* | *Characteristic(s)* |
| --- | --- | --- |
| ***acrD* knockout** |  |  |
| acrD_ko_fwd | TGTTGTGCTTAACCGGAGG | used to clone *acrD* knockout vector |
| acrD_ko_rev | AATGGTTGGGATCAGGGTG | used to clone *acrD* knockout vector |
| cat_out2 | CTTACGTGCCGATCAACG | reverse primer used to confirm insertion of Cm cassette into chromosomal *acrD* |
| cat_out3 | AGCATTCATCAGGCGGGC | reverse primer used to confirm insertion of Cm cassette into chromosomal *acrD* |
| cat_out4 | ACAAGGTGCTGATGCCGC | forward primer used to confirm insertion of Cm cassette into chromosomal *acrD* |
| cat_out5 | GTGATGGCTTCCATGTCG | forward primer used to confirm insertion of Cm cassette into chromosomal *acrD* |
| acrD_fwd | GAACGAACTGCAGACTGACAAGC | Primer flanking *acrD* knockout fragment (used to confirm Cm cassette insertion) |
| acrD_rev | GACAATGGTGACGGAGAACTGAC | Primer flanking *acrD* knockout fragment (used to confirm Cm cassette insertion) |
| ***acrD* overexpression** | |  |
| acrD-ApaI | ACAGGGCCCATGGCGAATTTTTTTATTGACCG | used to clone *acrD* overexpression vectors |
| acrD-SacI | AATGAGCTCTTAGTACGGCTTATCTTTTAGCG | used to clone *acrD* overexpression vectors |
| narP_ApaI | TATGGGCCCGCTTGCCATCCTCACC | used to clone upstream region of *acrD* |
| acrD_SalI | TCGGTCGACACAAACGCC | used to clone upstream region of *acrD* |
| ***baeR* overexpression** | |  |
| baeR_SacII | ATACCGCGGATGAACCAGATCCCCGCCAC | used to clone *baeR* overexpression vector |
| baeR_ApaI | GTAAAGCGGGGGCCCGGC | used to clone *baeR* overexpression vector |
| **Transcriptional promoter-*egfp* fusions** | |  |
| acrD_up | CGAACCCGAAGACTTGTTGG | used to amplify upstream region of *acrD* |
| acrD-P-egfp | CAGCTCCTCGCCCTTGCTCAGCATTTAAA  CAAAAACTCCACAGC | used to amplify upstream region of *acrD* (contains a 24-nt extension that is homologous to the start of the *egfp* gene) |
| acrAB_fwd | AAAGGTCATCGCATTGGCAT | used to amplify upstream region of *acrA* |
| acrA-P-egfp | CAGCTCCTCGCCCTTGCTCAGCATAAATAA  ACCTCGAATGTCCG | used to amplify upstream region of *acrA* (contains a 24-nt extension that is homologous to the start of the *egfp* gene) |
| egfp-ATG | ATGCTGAGCAAGGGCGAG | used to amplify the *egfp* gene flanked downstream by translational stop codons in all three reading frames and the transcriptional terminator t0 from phage λ |
| egfp-Cm | TACGCAAACCGCCTCTCC | used to amplify the *egfp* gene flanked downstream by translational stop codons in all three reading frames and the transcriptional terminator t0 from phage λ |
| acrD-P-fwd-SacII-2 | ATATCCGCGGCAACCGTACTCTGGC | nested primer used for fusion of the *acrD* promoter to the *egfp* gene |
| acrA-P-fwd-SacII | ATACCGCGGAGCGGTATGATTTACAACG | nested primer used for fusion of the *acrA* promoter to the *egfp* gene |
| uidA-t0-KpnI | TATGGTACCAACGGTGGTATATCC | nested primer used for fusion of a promoter region to the *egfp* gene |
| **Electrophoretic mobility shift assay** | |  |
| acrA-P-fwd2 | TGTTTGGTATTTCGTGCC | used to amplify *acrAB* promoter region |
| acrA-P-rev2-Cy5 | CTGAAAGCATCAGAACGG | used to amplify *acrAB* promoter region, Cy5 labeled |
| acrD-P-fwd2 | TCTGGCTGGAATTCTGTC | used to amplify *acrD* promoter region |
| acrD-P-rev2-Cy5 | AATCGCTAATACCCAGGC | used to amplify *acrD* promoter region, Cy5 labeled |
| tolC-P-fwd | GACCGCAGTGACCAATTA | used to amplify *tolC* promoter region |
| tolC-P-rev | TGGCTGGCAACACTGAAG | used to amplify *tolC* promoter region, Cy5 labeled |
| baeR_NcoI | TATCCATGGACCAGATCCCCGCCACTC | used to clone *baeR* into C-terminal His-tag protein expression vector |
| baeR_EcoRI | ATTGAATTCGGGATCAGACGACAGCCATC | used to clone *baeR* into C-terminal His-tag protein expression vector |
| **Quantitative RT-PCR** |  |  |
| acrA_RT_fwd | GCTTTCAGGGAGCTTAGC |  |
| acrA_RT_rev | ACTTCTGCGACTCGGAAC |  |
| acrD_RT_fwd | ATATCCCGATCTGGCTCCG |  |
| acrD_RT_rev | AAGTCAGGCTAACGGTGG |  |
| hrpL_RT_fwd | TATTCCGTGAGCATGGGC |  |
| hrpL_RT_rev | GCAATGCCAAACACCCAGG |  |
| recA_RT_fwd | TAAGGGCTCCATCATGCGC |  |
| recA_RT_rev | ACCTGCAAAGTCAGGGTGG |  |
